# Wellmap: a file format for microplate layouts

**DOI:** 10.1186/s13104-021-05573-0

**Published:** 2021-05-01

**Authors:** Kale Kundert

**Affiliations:** Wyss Institute for Biologically Inspired Engineering, Harvard University, Boston, MA 02138 USA

**Keywords:** File format, Microplate, 24-well, 96-well, 384-well, Python, R, TOML

## Abstract

**Objective:**

Microplates are ubiquitous in biological research because they make it easy to collect data for hundreds of different conditions in a single experiment. Despite this, there is no standard method to annotate the wealth of data contained in each plate.

**Results:**

We introduce a new file format, called wellmap, for describing the layout of wells on microplates. The format is text-based and emphasizes being easy to read, write, and share. It is capable of describing any layout for any experiment. It is also accompanied by a tool for generating clear visualizations of layout files, and a simple API for parsing layout files in analysis scripts written in python or R. We have used wellmap in our own research to annotate data from a wide variety of experiments, including qPCR and flow cytometry. Given the large number of experiments that make use of microplates, it is our hope that other researchers will find this file format as useful as we have. For complete instructions on how to install and use wellmap, visit: https://wellmap.rtfd.io.

## Introduction

24-, 96-, and 384-well plates are ubiquitous in biological research because they make it easy to collect data for hundreds of different conditions in a single experiment. Once the data have been collected, though, annotating which conditions were tested in which wells is more of a challenge than it might seem. These annotations must be easy to create, because a typical scientist might perform several microplate experiments every day. They must be easy to check for mistakes, because mislabeling the data could spoil the entire experiment. They must be easy to understand, because others might need to interpret the data without the help of the original scientist. And they must be easy to incorporate into analysis scripts, because the purpose of annotating data is to analyze them.

In the absence of a standard way to annotate microplate data, a number of ad hoc approaches have come into wide use. Unfortunately, none satisfy all the criteria listed above. Perhaps the worst approach is to describe plate layouts in a paper lab notebook. Such descriptions are easy to create, but hard to share, hard to keep associated with the data, hard to check for omissions or ambiguities, and impossible to use directly in analysis scripts. Another flawed approach is to hard-code layout information directly into analysis scripts. These scripts are both hard to write and hard for others to understand. They also encourage copy-and-pasting to perform similar analyses on different layouts, which makes it harder to fix bugs or otherwise improve the scripts. A better approach is to record layouts using spreadsheet files (e.g. CSV, TSV, XLSX). These files are easy to understand, and can be stored alongside the data so that they are unlikely to be lost. That said, spreadsheets are highly redundant because each condition must be specified separately for each well it applies to. This redundancy makes spreadsheets both tedious to create and prone to mistakes. It is also not trivial to parse annotations from a spreadsheet and associate them with raw data for analysis, although there are tools such as plater [1] or plate_maps [2] that can make this easier. Finally, some instruments come with proprietary software that can be used to specify plate layouts for experiments involving that instrument. These programs usually make it easy to create and visually check layouts, but they suffer from a lack of flexibility. Analysis is typically limited to a handful of options pre-programmed into the software, sharing layouts may require others to purchase the same software, some programs have arbitrary limits on the number of conditions per well or plates per analysis, and of course, these programs are not available for all instruments or all experiments. Overall, there is a need for a better way to annotate microplate data.

Here we address this need by introducing a file format, called wellmap, that can be used to describe any layout for any kind of microplate experiment. This file format is easy to write: The syntax is based on the established TOML configuration file format [3], and can be learned from just a few examples. There is minimal redundancy, so even complex layouts can be described succinctly. The file format is easy to check for mistakes: A program is provided to create visual maps of wellmap files, where any mistakes will stand out. The file format is easy to share: It is a text-based format, so no special software is required to read or write it. Additionally, the syntax is well-documented and intuitive enough that it can be understood even by those unfamiliar with it. Finally, the file format is easy to parse: Parsers are provided for python and R, the two most common languages used for the analysis of biological data. In both languages, the parsed layout is returned as a tidy [4] data frame. We hope that the wellmap file format will replace the existing methods for annotating microplate data and thereby make these experiments easier to annotate and analyze.

## Main text

### Workflow

The wellmap workflow has two steps. The first is to write a file that describes the plate layout for a particular experiment, and the second is to write an analysis script that makes use of the information in said layout file. Key aspects of both steps are highlighted below. For more in-depth and up-to-date information, refer to the online documentation: https://wellmap.rtfd.io.

#### Creating wellmap files

**Fig. 1:**
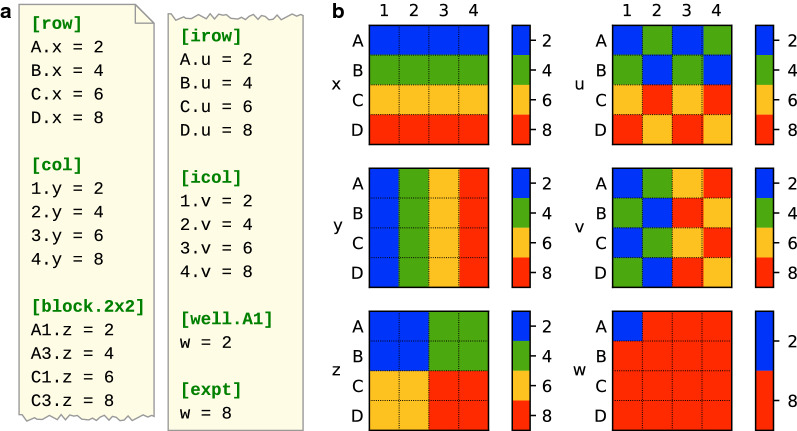
A demonstration of the most commonly-used well groups. **a** A contrived layout that uses seven different well groups. Each row specifies a different value for the “x” parameter, each column a different value for the “y” parameter, etc. **b** A visualization of the layout in **a**, as rendered by the wellmap command-line program. In each heatmap, the cells represent different wells and the colors represent different values of the parameter indicated on the y-axis

Wellmap files are organized into groups of wells, such as “row A”, “columns 3–5”, “a 2 × 2 block with A1 in the top left”, etc. Each well group can be associated with any number of experimental parameters, such as “mutant: Y37A”, “concentration: $100\,\upmu \hbox {g}/\hbox {mL}$”, “timepoint: 30 min”, etc. For example, the following snippet specifies that row A contains the Y37A mutant: 
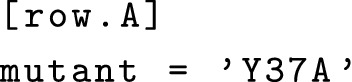


The wellmap file format is based on TOML [3], a format that emphasizes being easy to read and write by hand. Typically, square brackets are used to identify groups of wells and any “key = value” lines that follow are used to specify experimental parameters for those wells. Note however that all of the following are equivalent: 
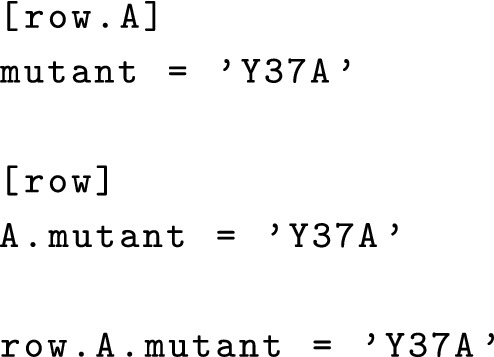


Figure 1 demonstrates most of the well groups that can be used in a wellmap file. Rows, columns, and blocks are particularly useful. Although not shown here, wellmap also supports (i) specifying layouts that span multiple plates, (ii) reusing information between related layouts, (iii) annotating layouts with metadata such as the name of the experimenter or the path to the raw data. See the online documentation for more information: https://wellmap.rtfd.io.

**Fig. 2:**
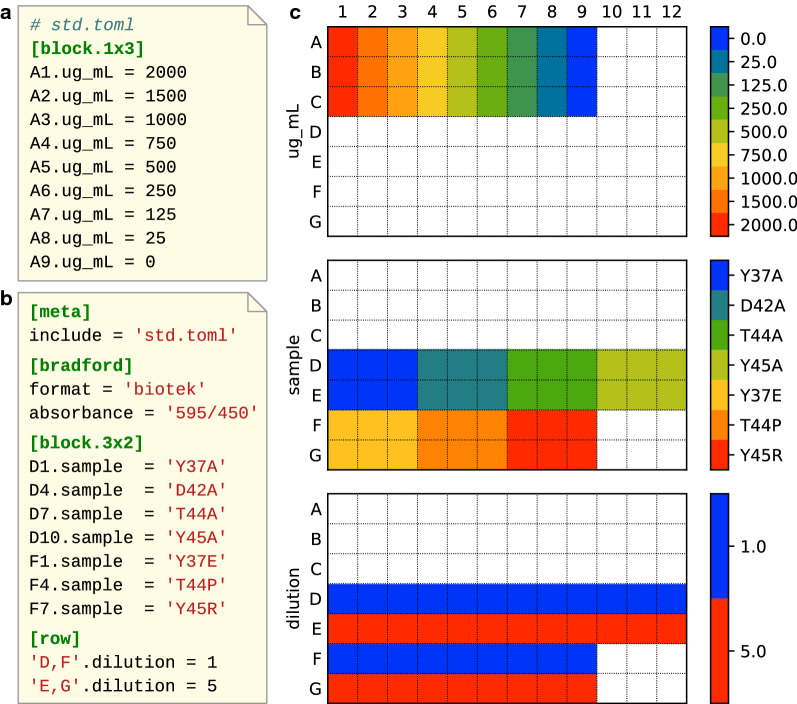
A real-life example of a layout used for a Bradford assay. **a** A layout file describing just the concentrations of a standard curve, which may be relevant to many layouts. These concentrations come specifically from the Pierce BCA Protein Assay Kit (ThermoFisher #23225). **b** A layout file describing the assay itself, including which mutants are being tested and what dilutions are being used. **c** A visualization of the layout in **b**, as rendered by the wellmap command-line program

Once a layout file has been written, it is important to double-check that it does not contain any mistakes. The best way to do this is to look at a visualization of the layout, such as those in Figs. 1 and 2. These visualizations were generated by a command-line program that is distributed with wellmap. Installing and using this program is quite simple: 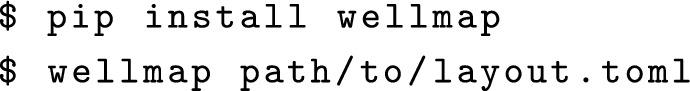


#### Parsing wellmap files

An important feature of the wellmap file format is that it can be easily parsed for use in analysis scripts. The API, which is available for both python and R, consists of a single load() function that parses a layout file and returns a tidy [4] data frame with a row for each well and a column for each experimental parameter. This data frame can subsequently be merged with unlabeled data to label each datum with the parameters specified in the layout file. The load() function can also perform this merge automatically, if requested.

In many cases, converting an existing analysis script to use wellmap will take little effort and will make the script both simpler and more powerful. Little effort because the wellmap API uses standard data types and follows the “Unix philosophy” of doing just one thing (and doing it well) [5]. Simpler because any code that was previously devoted to determining layout information can be replaced with a single command to parse a wellmap file. More powerful because the script will be able to analyze any layout that provides the expected experimental parameters, regardless of how the wells are organized on the plate.

### Discussion

Any project that uses scripts to analyze microplate data can benefit from wellmap. The file format itself is not specific to any particular experiment; it simply describes how wells are organized on plates. In our own research, we have applied wellmap to layouts from a broad range of experiments including quantitative polymerase chain reaction (qPCR), flow cytometry, enzyme-linked immunosorbent assays (ELISA), Bradford assays, Miller assays, and minimum inhibitory concentration (MIC) assays. Furthermore, incorporating wellmap into a project is not an onerous process. There are just two steps, both compatible with any workflow. First, write a short text file describing the layout of each experiment. Second, adapt any analysis scripts to load information from said files using a simple API.

There are several noteworthy advantages to using wellmap instead of an ad hoc approach to record plate layouts. The first is that wellmap makes it easier to write robust, reusable analysis scripts. Because all of the layout information is specified externally and parsed into a consistent format, the same analysis script will work seamlessly for any layout. And because the layout file can contain all the information needed for the analysis (even information that isn’t associated with particular wells, like the paths to the raw data files), the analysis will be easy to reproduce.

The second advantage is that the wellmap file format encourages good data management practices. A key principle of data management is that data should be maintained in a state where anyone (especially those not familiar with the project) could understand it. Wellmap is consistent with this principle because the file format is intuitive, easy-to-read, open-source, and well-documented. Another key principle of data management is that metadata should be kept in the same place as the data they describe, so that the two are less likely to be separated. Wellmap files are simple text files that can easily be stored alongside the data, and that can also specify the path(s) to the data file(s) in question. Both of these factors establish a strong link between the data and the metadata.

The third advantage is that several features of the wellmap file format combine to defend against mistakes. Foremost of these features is the ability to generate clear visual maps of layouts to check for mistakes. But also important is the fact that the file format avoids redundancy. When each piece of information is only specified in one place, errors are both harder to make and easier to fix. Similarly, using a single layout file for both note-keeping and analysis eliminates the possibility of there being discrepancies between the two.

Other packages have been developed to help analyze microplate data, but wellmap is the only one that provides a file format specifically designed for this task. Most of these alternatives use spreadsheet files, which are disadvantageous for the reasons discussed in the introduction. plater is an R library for parsing plate layouts from spreadsheet files into tidy data frames [1]. It has excellent documentation and is easy to use, but is only available for R (not python) and does not provide a graphical tool for visualizing plate layouts. plate_maps is a command-line script for converting plate layouts stored in spreadsheet files into tidy CSV or TSV files [2]. It can be used with any scripting language, but cannot visualize layouts or merge layouts with experimental data. Several other packages have the ability to load plate layouts from files (mostly spreadsheets), but do not make that information readily available to analysis scripts [6–8]. Mostly these packages have some other focus, such as analyzing data from RNAi experiments [6] or simulating robotic pipetting protocols [7].

As a demonstration of everything described here, Fig. 2 shows a wellmap file that was used in a real experiment. In this case, the experiment is a Bradford assay [9] meant to measure the concentrations of several purified protein mutants. This layout has several notable features. The first is that the standard curve is factored into its own file, so that it can be reused between experiments. This will make it especially easy to specify layouts for future Bradford assays. The second is that the layout seamlessly combines row-oriented, column-oriented, and block-oriented features. The “columns” in the standard curve are actually 1-by-3 blocks—because columns grow to fill all available rows, and the standard curve is meant to be included in layouts with any number of rows—but it is clear that the file format can succinctly describe layouts with realistic levels of complexity. Third, the [bradford] section provides information on how to parse and interpret the data, namely what format the data is in (since different plate readers export data in different formats) and what wavelengths were measured. With this information, the layout file contains all the information needed to analyze the results of the experiment.

### Conclusion

The wellmap file format provides an improved way to annotate data from microplate experiments, which are ubiquitous in biological research. Plate layouts are described using simple text files, which can quickly be written and readily be understood. These files can then be directly used for both visualization and analysis. Incorporating wellmap into an analysis pipeline is straight-forward and offers benefits ranging from greater flexibility to better data management. In summary, we hope that this software will be broadly useful to the large community of scientists who routinely perform microplate experiments.

## Limitations

- Using wellmap requires the ability to program in either python or R. For this reason, wellmap will be most useful to researchers who are already in the habit of writing their own analysis scripts for microplate experiments.
- There are currently no third-party packages that use wellmap to analyze data from specific kinds of experiments (e.g. qPCR) or instruments (e.g. plate readers). Such packages would make it easier to get started with wellmap, especially for non-programmers. We have plans to develop some packages like these in the future, though.

## Data Availability

Project name: wellmap Project home page: https://wellmap.rtfd.io/ Operating system(s): Platform independent Programming language: Python, R Other requirements: Python>=3.6 or R>=3.0 License: MIT Any restrictions to use by non-academics: No.

## Abbreviations

API: Application programming interface
CSV: Comma-separated values
TSV: Tab-separated values
XLSX: Microsoft Excel open XML spreadsheet
TOML: Tom’s obvious minimal language
